# Mathematical comparison of protocols for adapting a bacteriophage to a new host

**DOI:** 10.1093/ve/veae100

**Published:** 2024-11-22

**Authors:** James J Bull, Stephen M Krone

**Affiliations:** Department of Biological Sciences, University of Idaho, 875 Perimeter drive, Moscow, ID 83844, United States; Institute for Modeling Collaboration and Innovation, University of Idaho, 875 Perimeter drive, Moscow, ID 83844, United States; Institute for Modeling Collaboration and Innovation, University of Idaho, 875 Perimeter drive, Moscow, ID 83844, United States; Department of Mathematics and Statistical Science, University of Idaho, 875 Perimeter drive, Moscow, ID 83844, United States

**Keywords:** phage therapy, computational model, protocol, mathematical model

## Abstract

Interest in phage therapy—the use of bacterial viruses to treat infections—has increased recently because of the rise of infections with antibiotic-resistant bacteria and the failure to develop new antibiotics to treat those infections. Phages have shown therapeutic promise in recent work, and successful treatment minimally requires giving the patient a phage that will grow on their infecting bacterium. Although nature offers a bountiful and diverse supply of phages, there have been a surprising number of patient infections that could not be treated with phages because no suitable phage was found to kill the patient’s bacterium. Here, we develop computational models to analyze an alternative approach to obtaining phages with new host ranges—directed evolution via laboratory propagation of phages to select mutants that can grow on a new host. The models separately explore alternative directed evolution protocols for phage variants that overcome three types of bacterial blocks to phage growth: a block in adsorption, temperate phage immunity to superinfection, and abortive infection. Protocols assume serial transfer to amplify pre-existing, small-effect mutants that are initially rare. Best protocols are sensitive to the nature of the block, and the models provide several insights for enhancing success specific to each case. A common result is that low dilution rates between transfers are beneficial in reducing the mutant growth rate needed to ascend. Selection to overcome an adsorption block is insensitive to many protocol variations but benefits from long selection times between transfers. A temperate phage selected to grow on its lysogens can evolve in any of three phenotypes, but a common protocol favors the desired changes in all three. Abortive infection appears to be the least amenable to evolving phage growth because it is prone to select phages that avoid infection.

## 1. Introduction

An ongoing rise in drug-resistant bacterial infections has motivated attempts to revive the use of bacteriophages to treat infections (phage therapy, D’Herelle 1926, Rhoads et al. 2009, Wright et al. 2009, Gill and Young 2011, Chan et al. 2013, Sarker et al. 2016, Schooley et al. 2017, Abedon 2018, Dedrick et al. 2019, 2021, 2023, El Haddad et al. 2019, Kortright et al. 2019, Hatfull 2023, Pirnay et al. 2024). Both historically and as currently practiced, phage therapy is often a matter of administering to the patient any “lytic” phage that grows on the infecting bacterium. Despite the extreme abundance of diverse phages in nature, a surprisingly common rate-limiting step in phage therapy is obtaining environmental phages that infect a patient’s bacterium (Schooley et al. 2017, Dedrick et al. 2019, 2021, 2023, Pirnay et al. 2024). The problem is that bacteriophages mostly have narrow or idiosyncratic host ranges, infecting a small subset of the strains within a bacterial species or genus. Thus, the phage strains that kill the bacteria of one patient’s infection often will not work on the bacteria of another infection, even bacteria of the same species. Relying on environmental sampling to obtain the needed phages is one approach, but “directed” laboratory evolution of phage host range offers a potential alternative that is sometimes—if not often—successful in changing the host range of a phage (Adams 1959, Hashemolhosseine et al. 1994, Hashemolhosseini et al. 1994, Meyer et al. 2012, Mapes et al. 2016, Burrowes et al. 2019). Yet directed evolution is often applied without awareness of which protocols are most likely to yield the desired outcome. Failures of directed evolution are not likely to be reported, creating an ascertainment bias in the literature against cases that might have benefitted from a better protocol.

There is a long history of studying the genetic and molecular bases of phage host range determination, the corresponding bacterial blocks to phage infection, and the types of changes in phages that enable them to overcome the block (Adams 1959, Hashemolhosseine et al. 1994, Hashemolhosseini et al. 1994, Crill et al. 2000, Poullain et al. 2008, Hyman and Abedon 2010, Labrie et al. 2010, Born et al. 2014, 2014, Mapes et al. 2016, Burrowes et al. 2019, de Jonge et al. 2019, Sergueev et al. 2019, Yehl et al. 2019, de Leeuw et al. 2020, Borin et al. 2021, Huss et al. 2021, Latka et al. 2021, Versoza and Pfeifer 2022). The most common approach of directed evolution is to plate a large number of phages on the new, initially nonpermissive host and look for plaques; such plaques are usually due to a single-step, large-effect mutation that overcomes the host block. However, when large-effect mutations are not accessible, the evolution of acceptable levels of phage growth on a new host may require an accumulation of small-effect mutations, such as being selected by serial propagation on the new host (Crill et al. 2000, Hall et al. 2011, Nguyen et al. 2012, Borin et al. 2021, Kok et al. 2023). For the gradual evolution of a change in host range, there are many variations of protocol that may be entertained, and it is not necessarily intuitive which of those variations are best. Gradual evolution is challenging because it is blind—it requires applying a selective protocol without evidence that the phage has achieved possible incremental growth on the nonpermissive host.

Here, we use computational models of phage–bacterial dynamics to evaluate the consequences of variations in protocol to evolve a phage to grow on a nonpermissive host. If mutations are readily available that allow the phage to form a plaque on the new host, then the evolution is trivial. But when multiple mutations are required to attain good phage growth, the protocol will matter. The purpose here is to understand which protocols are likely to offer the best and fastest selection of increases in phage host range via small-effect mutations. The models are agnostic to whether the mutations arise naturally, are introduced from a recombinant pool of different phages, or are from an engineered library of mutants. By itself, the evolution of any single, small-effect mutation may not be sufficient to yield a phage with sufficient growth on the nonpermissive host to be useful in therapy, but sequential evolution of multiple small-effect mutations may eventually yield a suitable phage. We limit the analysis to one phage that grows well on a permissive host but initially fails to grow at all on a nonpermissive host, and we mathematically introduce mutants of that phage that have specific properties relevant to growth on the nonpermissive host. We apply these models to three different types of host blocks to phage growth: the bacterium blocks phage adsorption, bacterial lysogens kill their superinfecting temperate phages, and the bacterium allows phage infection but aborts/kills the phage after infection.

To anticipate the overall message from this study, our goal is to provide heuristic principles of directed evolution that can be used empirically across diverse systems. Computational analysis of mathematical models is used to guide intuition, but despite the quantitative output, the results are interpreted qualitatively. The models can neither capture all relevant details of any system nor span the realm of possible protocol variations, but they can suggest principles that may apply widely and without the need for specifying every minute detail of an implementation. A foundation of our study is to compare variations in protocol to identify whether the evolution is strongly affected. Although empirical research could test the relative merits of those alternatives, it is far easier to compare protocols with numerical analyses than experimentally. When the apparent superiority of a protocol feature is supported by models and intuition, the user may merely wish to adopt the purported best practice. It is also obvious that any attempt at directed evolution ultimately depends on the mutation supply; methods to increase the input of mutations can help up to a point, but those methods lie outside of our study.

## 2. Materials and methods

### 2.1. Biological justification of the modeling approach

At a coarse level, adapting a phage to a new host is merely a matter of mixing the phage with that host and providing an opportunity for growth. Mutants that can overcome the block will grow, thereby increasing their frequency in the background of the wild type. But when those mutants are only slightly able to grow, plaques and other easily-recognized indicators of growth may be hard to detect. Detection of a small-effect growth mutant will generally not be possible until it has evolved to a high abundance in the culture or until multiple beneficial mutations have accumulated. In these cases, there are then several properties of the protocol being used with subtle but potentially important consequences.

We will assume throughout that there is a common wild-type phage in a population that also carries one or two rare mutants. To grow the phages, the culture also contains two bacterial hosts, permissive and nonpermissive (in some protocols, the cultures alternate between permissive and nonpermissive hosts); the permissive host is needed to amplify the phages. In most protocols, the phages are subjected to serial transfer: the phages and bacteria are added to a liquid culture and grown for a fixed-interval “cycle” (Bull et al. 2011). A sample of the culture (containing free phage, infected cells, and uninfected cells) is added to a new liquid culture with bacteria at a predetermined density. This transfer protocol may be continued indefinitely, but here we fix the duration to facilitate comparisons and to mimic a protocol that would be practical when attempting to evolve a phage on demand. Thus, different trials are typically terminated at 10 h (and at the end of a cycle) so that any differences in evolution can be attributed to the protocol structure, not to different durations of evolution or to different stages of a cycle. Serial transfer using growth on plates may also be appropriate for directed evolution of phages, but accurately modeling plate growth is more complicated than liquid. Nonetheless, many protocol properties that apply to liquid growth will apply to plate growth.

With any protocol, there are obvious needs such as appropriate media, temperature, level of aeration, and bacterial state of growth. These will often be specific to the bacteria and phage being used. Instead, our focus is on protocol properties that influence the dynamical properties of the culture and host presentation, whose consequences are easily modeled and transcend details specific to individual systems. These protocol matters generally fall under the realms of “how much, of what, and how long?” They are well suited to modeling. More details about the specific topics will be provided below.

### 2.2. Basic model structure

The data presented in this study were generated by numerical analysis of ordinary differential equations using NDSolve in Mathematica® (v. 13.3.0.0) and plotted using Mathematica. (Although Mathematica is proprietary, free versions are available that may be used to run the programs provided in our Supplementary material.) To anticipate the formal equations presented later, we explain the general nature of them here. The equations track free bacterial densities, free phage densities, and infection densities in single cultures. Free bacteria (*B*) are assumed to grow and be depleted only through phage infection until the culture is terminated:

Changes to *B* = gains from growth − losses from phage infection.

In most models, there are two bacterial strains: the permissive strain *B*_1_ that supports the growth of the wild-type phage and possibly the mutant phages, and the nonpermissive strain *B*_2_ that only the mutant phage can productively infect. (We use the same symbol for the strain name and for its density.)

Each phage type is described by two equations. One equation tracks the fate of free phage (density *P*); the free state is “lost” upon infection of cells and gained by bursts of infected cells. The other equation tracks infected cells (density *I*), a state which is gained when free phages infect cells and lost when the cells burst to release phage progeny:

Changes to *P* = gains from bursts of infected cells − losses to infection of free cells.
Changes to *I* = gains from infection of free cells − losses to bursts.

We will designate by *P*_1_ the wild type and *P*_2_ the mutant (*P*_3_ is a second type of mutant in one model).

We note that these equations result in an exponential distribution of lysis times, which is biologically unrealistic. Supplementary S1 compares results among different ways of modeling lysis time and finds that the approach we use agrees well with more realistic assumptions. The method we use allows computational simplicity.

These equations apply to growth in a single culture that will typically span a “cycle” of 20–90 min—or longer—depending on bacterial growth rate and phage killing. The attempt to evolve growth on a new host will often involve serial transfer across multiple cultures. To model serial transfer, the code assumes that the first culture is initiated with bacteria and phage. The culture is grown for a set number of minutes (specified as “cycle length” in the protocol), then an aliquot of the free phage plus cells (whether infected or not) is added to the next culture of new cells at the specified density. The relative volume of the aliquot (hence the number of phage carried over) is specified by the dilution.

Free phage densities are measured at the end of the trial (typically 10 h). The models make no assumptions about how the mutant density is observed throughout the process. Indeed, one benefit of the modeling approach is that it can inform how to best select phage mutants even when the first mutations to accumulate are not empirically assayed. Ultimately, if the evolution is to be of practical benefit, the final, evolved phage must grow well enough on the new host to form plaques, clear cultures, or generally suppress bacterial numbers. But the goal of the modeling is to identify procedures fostering the ultimate evolution of useful phages for which the intermediates might not be detected.

Different sets of equations apply to each type of bacterial block to phage growth, as given in their respective sections later. As explained earlier, the numerical trials are not run to equilibrium but instead run for a fixed interval of typically 10 h. Use of a continuous culture “chemostat” to evolve phage host range would allow for much longer growth per experimenter effort (Meyer et al. 2012), but chemostats are not practical for some of the protocol variations considered here. We also acknowledge that the cycle times simulated here are much shorter than would be appropriate for slowly growing bacteria (e.g. *Mycobacterium*), and to accommodate such cases, the equations would need to be reparameterized. We expect, however, that the qualitative outcomes observed here will generalize.

A critical step in evolution is the origin of an appropriate mutant. Mutation origin will often be a step that involves chance (randomness), and it may have a long waiting time. In general, larger populations decrease the waiting time. However, our interest here is in how a protocol affects evolution after a mutation not only has arisen but is also present in sufficient numbers that randomness can be ignored (we assume deterministic processes, albeit starting with a low density of the mutant). Deterministic models of phage evolution can be developed either as genotype frequencies or as genotype densities. We use densities on the grounds that any mutant capable of growth on the nonpermissive host will be easy to isolate if it reaches high abundance, regardless of its frequency relative to the wild type.

## 3. Results

We consider the three different blocks to phage infection separately. Although the protocols for evolving phage to overcome these blocks all involve serial transfer, the details for best practices within serial transfer differ, necessitating separate analyses.

### 3.1. Model 1: the nonpermissive host blocks adsorption

This block to infection is perhaps the most common one considered: the phage cannot grow on the nonpermissive host because it cannot adsorb/attach to and get inside that host (Adams 1959, Hashemolhosseine et al. 1994, Hashemolhosseini et al. 1994, Borin et al. 2021). If the genome enters the cell, the phage life cycle progresses normally. Evolution to grow on the new host is thus evolution of adsorption to that host.

The following protocol issues need to be decided:

- Should the phage be grown in a mix of the permissive and nonpermissive host, or be grown on just the nonpermissive host with occasional amplification on the permissive host? Following the work of Bull et al. (2022), we will refer to these alternatives as Mixed versus Sequential host presentation. Mixed refers to growth in the presence of both hosts, and Sequential is an alternation.
- Does growth on the permissive host provide any benefit other than ensuring that the phage population does not die out?
- What dilutions should be used? Serial transfer requires that a subsample (aliquot) of one culture be transferred to the next culture unless the entire phage population can be concentrated into a small volume free of expended media and bacterial excretions. Practical aliquots for transfer range from 0.1 of the original volume to 10^−4^ of the original volume. Larger aliquots result in more phage being transferred and will more quickly exhaust the permissive host.
- How long should cultures be grown before transfer? Longer cycles will allow more phage growth until host exhaustion; in mixed cultures, duration will affect the balance of phage growth on the permissive host versus the nonpermissive host because the permissive host will be exhausted first.

Answers to some of these questions seem intuitive. For example, dilution should always be bad for evolution because it reduces phage numbers from one cycle to the next, thereby requiring enough growth to offset the loss; dilution is merely a necessary evil of transferring suspended phages that have not been concentrated. Conversely, longer cycles should be better for evolution because there is more opportunity for mutant phage growth on the nonpermissive host, at least to the point that those bacteria are exhausted. Somewhat less intuitive is the benefit of adding the permissive host when selecting for growth on the nonpermissive host: is there a benefit of permissive host levels above that needed to maintain the phage population? Could growth on the permissive host actually be counterproductive? Likewise, it is not immediately obvious whether there is much of a difference between Mixed and Sequential host presentation. Even if the qualitative effects of some of these properties seem obvious, initial intuition can fail, which is why we use the models. We address these questions both individually and in combination.

#### 3.1.1. The model

We offer the equations here, but the reader is assured that the paper can be understood without recourse to them. The notation given in Table 1 is perhaps useful for following some of the text:


(1) $$\begin{array}{l} B_{1}^{^{\prime}} = \,{B_1}\left( {r - {k_{11}}{P_1} - {k_{21}}{P_2}} \right)\\ B_{2}^{^{\prime}} = {B_2}\,\left( {r - {k_{22}}{P_2}} \right)\\ P_{1}^{^{\prime}} = {b_{11}} \delta_{11} I_{11} - k_{11}P_1B_1 \\ I_{11}^{^{\prime}} = {k_{11}}{P_1}\,{B_1} - \,{\delta _{11}}{I_{11}}\\ P_2^{^{\prime}} = {b_{22}}{\delta _{22}}{I_{22}} + {b_{21}}{\delta _{21}}{I_{21}} - {k_{22}}{P_2}{B_2} - {k_{21}}{P_2}{B_1}\\ I_{22}^{^{\prime}} = {k_{22}}{P_2}{B_2} - {\delta _{22}}{I_{22}}\\ I_{21}^{^{\prime}} = {k_{21}}{P_2}{B_1} - {\delta _{21}}{I_{21}} \end{array}$$

**Table 1.** Notation for Model 1 (adsorption block).

Terms	Meaning	Values used (units)
Variables (functions of time)		
*B_i_*	Uninfected density of bacterium *i* (*i *= 1, 2). *B*_1_ is permissive, *B*_2_ is nonpermissive	Initial values: 10^8^ for each (/ml), except where specified
*P_i_*	Density of free phage *i* (*i *= 1, 2). *P*_1_ is the wild type, and *P*_2_ is the mutant	Initial value 10^8^ for *P*_1_; 1 for *P*_2_ (/ml), the latter at 8 min
*I_ij_*	Density of “infected” bacterium *ij* (phage *i* in bacterium *j* (*i* = 1, 2, 3; *j* = 1, 2))	Initial values 0 (/ml)
Parameters		
*r*	Bacterial growth rate	0.02 (/min)
*k_ij_*	Adsorption rate constant of phage *i* on cell *j*	≤10^−9^ (ml/min)
*δ_ij_*	Rate constant of transition from infected bacterium (*ij*) to burst	0.05 (/min)
*b_ij_*	Phage number of progeny from *I_ij_*	50 (individuals)
*C* ^a^	Cycle length	50 (min)
Dilution^a^	Fraction of free phage transferred at cycle end	0.1

a These parameters are absent from Equation (1) but included in the code.

These equations apply from the start of a “cycle” until its end, whence a fraction of the culture (consisting of free phage, infected cells and uninfected cells) is transferred to a new culture with free bacteria at the requisite densities and the infections start anew; the new cells are added at the concentration given but in an amount that is discounted by the amount of the old culture carried over. There are thus three other properties of the protocol that must be specified: the “dilution” gives the fraction of the old culture carried over to the new culture; the “cycle length” gives the number of minutes between start of a new culture and its transfer to the next; host presentation (Mixed or Sequential) determines whether each cycle starts with a single bacterial strain or both. The codes thus include more steps than shown in Equation (1).

**Figure 1.**
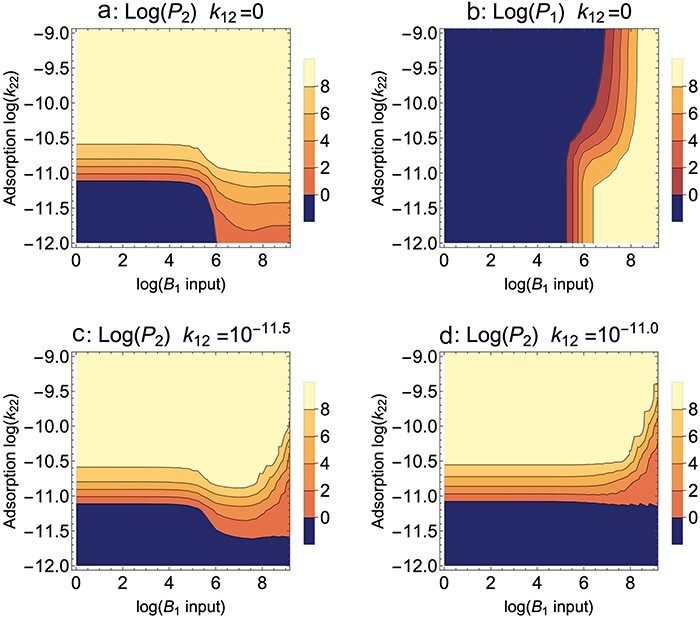
*B*_2_ blocks adsorption, Mixed presentation. Contour plots of the densities of the mutant phage (*P*_2_) and wild type (*P*_1_) after 10 h of propagation; the color key gives log_10_ densities of the phage indicated by the panel title (/ml). The fate of a single phage is shown in separate panels as a property of permissive host input to each cycle (*B*_1_, *X*-axis) and of the adsorption rate of the mutant phage to the nonpermissive host (*k*_22_, *Y*-axis). The wild-type phage does not adsorb to the nonpermissive host in (a) and (b), hence *k*_12_ = 0. (a) For adsorption rates >10^−10.5^, the mutant ascends regardless of *B*_1_ input. At lower adsorption rates, the mutant benefits from high *B*_1_ input. (b) Maintenance of the wild-type phage is affected by competition from the mutant phage. The lower part of the panel (the smallest *k*_22_ values and high B_1_ input) shows maintenance of the wild-type phage in absence of strong competition from the mutant, because the mutant does not reach high values there (from (a)). High *k*_22_ values expand the zone of wild-type loss, due to competitive exclusion by the mutant phage. (c, d) The benefit of high *B*_1_ for mutant evolution fades and becomes detrimental if the wild-type phage grows partially on *B*_2_. *k*_12_ = 10^−11.5^ for (c). *k*_12_ = 10^−11.0^ for (d). Bacterial growth rate = 0.02/min, cycle length = 50 min, dilution = 1/10, all burst sizes = 50, all transition rates from the infected state to burst = 0.05/min, adsorption rates of both phages to the permissive host = 10^−9^ ml/min. Per cycle input of the nonpermissive host *B*_2_ is 10^8^; initial value of the wild-type phage = 10^8^ and that of the mutant phage = 1 (introduced at 8 min).

The equations exclude many possible biological details, such as intrinsic phage and bacterial death, superinfection, and logistic bacterial growth toward a carrying capacity. We have explored the consequences of some elaborations and found little qualitative effect for the parameter values and initial conditions used. We have opted for the simplest equations that capture the basic properties, but it may be desirable to include additional details when attempting to mimic a specific implementation. Supplementary S1 provides a comparison of our model of lysis times to more realistic models for the trials in Fig. 1a and b. The exponential model seems adequate.

#### 3.1.2. The effects of permissive host supplementation, dilution, and cycle length

These first analyses will assume Mixed host presentation, in which both the permissive host (*B*_1_) and the nonpermissive host (*B*_2_) are combined at the start of each cycle/culture. A single set of parameter values and initial values will be used throughout the paper, except where they are varied to study the effect of specific parameters or are specific to the type of nonpermissive block to phage reproduction. These values are given in Fig. 1 and Table 1; they are chosen to represent a moderately challenging case for selection of the mutant, a small step toward growth on the nonpermissive host. At the start of each trial, mutant phage numbers (*P*_2_) are always a factor of 10^−8^ less than those of the wild type (*P*_1_) and typically introduced at 8 min to allow the wild-type phage dynamics to settle; for most trials of Model 1, the mutant differs from the wild type only in its adsorption rate to the nonpermissive host (*k*_12_ = 0 for the wild type, *k*_22_ = 10^−11^ for the mutant unless it is varied across a range of values). Here, *k_ij_* indicates the adsorption rate constant of phage *i* on host *j*; more generally, if two subscript indices are used, the first indicates the phage and the second indicates the bacterium.

A simple protocol property to consider is how the level of permissive host added per cycle (*B*_1_) affects the evolution of the mutant phage, *P*_2_. This is a somewhat challenging empirical problem because the goal is to grow the “mutant” to high numbers, but the only practical measurement is of the entire phage population, which will be heavily dominated by the wild-type phage initially (recall that we are assuming that *P*_2_ cannot yet form plaques on the nonpermissive host). The models allow us to track the two phages separately and thereby determine if they are affected differently. Figure 1 is a contour plot showing the levels of mutant phage (*P*_2_, Fig. 1a) and wild-type phage (*P*_1_, Fig. 1b) while varying the amount of *B*_1_ added each cycle and varying the adsorption rate of the mutant on *B*_2_ (*k*_22_); the adsorption rate constant on *B*_1_ is the same for both phages (*k*_11_ = *k*_21_ = 10^−9^); and a later analysis will allow k_21_ to differ from k_11_ (Section 3.1.3).

Perhaps surprisingly, there is benefit of adding large amounts of *B*_1_ every cycle. Furthermore, this benefit is moderately abrupt—it is only manifest at *B*_1_ inputs of 10^6^ and higher and only matters for adsorption rate coefficients (*k*_22_) below ∼10^−10.5^. A comparison of Fig. 1a and b indicates that the failure of mutant ascent (at lower *B*_1_ input levels and the lowest adsorption rates) coincides with a failure to maintain both phages, not competitive exclusion, a conclusion that is also supported by inspection of time-course dynamics (not shown). This much is understandable: bacteriophage maintenance requires enough growth to offset dilution, and low inputs of the permissive host are not sufficient to overcome dilution. Although both phages adsorb well to *B*_1_, low *B*_1_ limits phage growth no matter what the adsorption rate. *P*_2_ has an additional boost from growth on *B*_2_, but when adsorption to that host is low, that boost is not sufficient to allow the phage to reach high levels in 10 h.

At first sight, there is an effect of *B*_1_ that is unintuitive: the highest levels of *B*_1_ actually augment the ascent of *P*_2_ (at low k_22_). We suggest that this is merely a manifestation of the preceding point. High *B*_1_ helps offset the loss from dilution, and the effect works for *P*_2_ just as it works for *P*_1_ because both phages grow well on *B*_1_. Furthermore, *P*_2_ benefits increasingly as the level of *B*_1_ input increases (as indicated by the light contour expanding into smaller k_22_ values as *B*_1_ increases). Yet this effect does not generalize. It applies most strongly if the wild-type phage fails to adsorb to the nonpermissive host (*k*_12_ = 0). As wild-type adsorption to *B*_2_ increases, even modestly, the mutant’s evolution no longer benefits from high *B*_1_ and even becomes suppressed (Fig. 1c and d). These results can perhaps be rationalized in hindsight, but they would have been difficult to anticipate without the models. Empirically, it would be necessary to confirm a virtually complete absence of wild-type growth on the nonpermissive host before adopting a protocol suggested by Fig. 1a.

We emphasize that the graphs (Fig. 1) should be interpreted at a broad scale and that the exact positions of the contours have little predictive value. Exact contour positions vary with the duration of the propagation (our use of 10 h is arbitrary), with the initial density of the mutant, and with many other details that are of no specific interest. What is of interest is that there are broad zones in which the mutant does not invade, and broad zones in which it does invade (dark blue represents a density of ≤1 phage/ml and thus a complete failure to evolve). Furthermore, mutant enhancement by a high input of the permissive host, even for very low adsorption rates (*k*_22_), is also a result that applies over a broad span of the parameter space—but dependent on the wild type being unable to grow on the nonpermissive host. These are the main conclusions to be drawn that have practical value.

**Figure 2.**
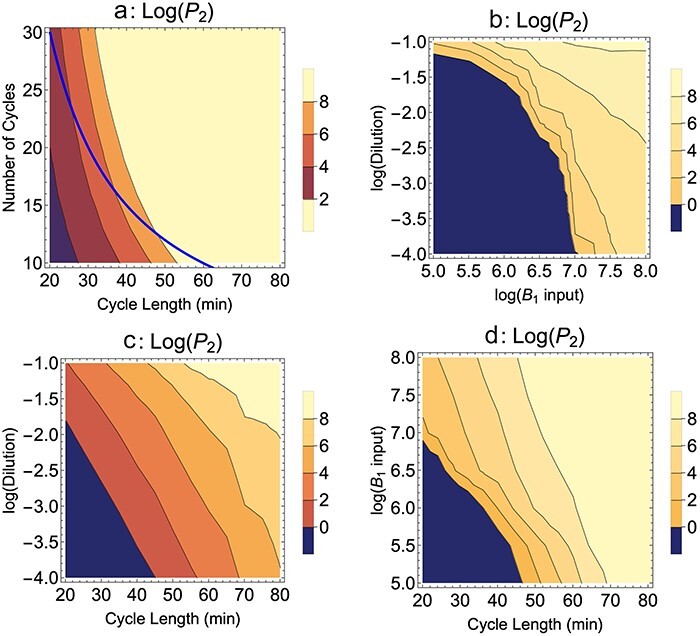
*B*_2_ blocks adsorption, Mixed presentation. The evolution of *P*_2_ levels after 10 h of serial transfer for various protocols varying the number of cycles, cycle length, dilution, and input level of *B*_1_. Note that (a) shows outcomes for shorter and longer durations of serial transfer than 10 h; 10 h applies to the points on the blue curve. The generalities are fewer dilutions, longer cycles, and larger inputs of bacterium *B*_1_ all benefit evolution of the mutant (all else equal). Parameter values and initial conditions are given in Fig. 1, except where varied on one of the axes. In (c) and (d), cycle length is varied, so the *P*_2_ value is taken within a minute of a cycle’s end once the serial transfer has exceeded 580 min—because 600 min need not correspond to a cycle’s end when cycle length is varied. Parameter values are as given in Fig. 1;*k*_22_ = 10^-11^. In all panels, the wild-type phage fails to adsorb to the nonpermissive host, *k*_12_ = 0.

As noted earlier, the effects of many protocol variations seem obvious. Figure 2 shows four different comparisons of dilution, cycle length, number of cycles, and addition of *B*_1_. There are no surprises: longer cycles are better, less dilution is better, and more *B*_1_ is better (as shown in Fig. 1). Figure 2a shows that, for the same total duration of selection, longer cycles are better, no doubt due largely to the fewer dilutions with fewer cycles (the data for 10 h of growth lie on the hyperbola shown).

Suppose a first mutation sweeps and a second beneficial mutation arises on that background. If the first mutant grows well enough that it can be maintained on the nonpermissive host, is there a continued benefit of adding the permissive host? For the limited trials explored here (which assume that the wild-type ads rate *k*_12_ is greater than 0), adding the permissive host exhibits a diminishing benefit as *k*_12_ increases (as suggested by Fig. 1). High *B*_1_ levels (e.g. in excess of 10^8^) even retard spread of the second mutation. Thus, at the point that the phage population can be maintained on just the nonpermissive host, the protocol should change to eliminate the use of the permissive host. Even if the mutant maintains itself on the pure nonpermissive host without clearing the culture or forming plaques, its maintenance can be confirmed by plating on the permissive host. It is perhaps unintuitive that high levels of the permissive host facilitate evolution of the first mutations but not of later ones.

#### 3.1.3. A trade-off in adsorption rates

**Figure 3.**
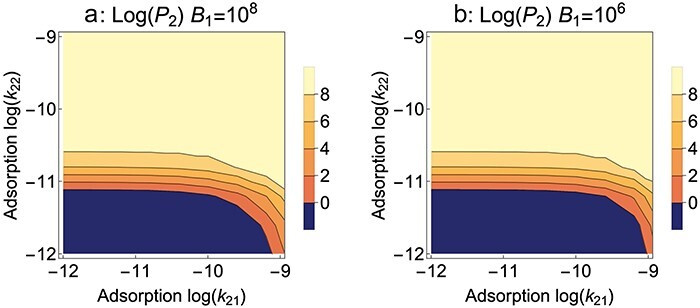
*B*_2_ blocks adsorption, Mixed presentation. Contour plots of mutant phage evolution after 10 h of serial transfer. In contrast to Figs 1 and 2, here the adsorption rate constants of the mutant phage are allowed to vary on both hosts (*k*_21_ on *B*_1_ and *k*_22_ on *B*_2_), and it becomes possible to evaluate the effect of a trade-off in adsorption rates. The wild type has values *k*_11_ = 10^−9^ and *k*_12_ = 0, so a trade-off requires that the mutant must have *k*_21_ < 10^−9^ for any increase in *k*_22_—its realm of possible values would lie inside the right boundary. It is thus seen that a strong trade-off can thwart mutant evolution because the mutant cannot evolve with strong reductions in *k*_21_ and only modest gains in *k*_22_ (dark blue zone). However, once the mutant’s adsorption rate on the nonpermissive host exceeds 10^−11^, reduced adsorption to the permissive host no longer suppresses its evolution. (a) and (b) differ only in the level of *B*_1_ input at the beginning of each cycle. There is only a slight effect of *B*_1_ input, indicated by the slightly expanded dark blue zone for 10^6^. (The right vertical boundaries of these plots correspond to slices at the appropriate *B*_1_ levels in Fig. 1a.) Parameter values are as given in Fig. 1; *k*_12_ = 0.

The benefit to the mutant from high permissive host levels (high *B*_1_) may depend on its adsorption rate to the permissive host. If adsorption to the nonpermissive host somewhat impairs adsorption to the permissive host (i.e. a trade-off), then adding high levels of the permissive host might offer more benefit to the wild type than to the mutant. Figure 3 shows contour plots that vary both mutant adsorption rates for each of two different input levels of *B*_1_: 10^8^ (Fig. 3a) and 10^6^ (Fig. 3b). Focus on Fig. 3a first. Since the wild-type adsorption rates are *k*_11_ = 10^−9^ and *k*_12_ = 0, a trade-off means that any increase in *k*_22_ above 0 by the mutant will reduce its *k*_21_ below 10^−9^. Thus the mutant’s options lie inside the right boundary of the figure, perhaps into the dark blue zone of no evolution if the mutant gains only a slight ability to infect the nonpermissive host. Only until *k*_22_ exceeds ∼10^−11^ does a reduced adsorption to *B*_1_ become irrelevant; however, if the trade-off is slight (*k*_21_ is only slightly reduced <10^−9^), then even slight increases in *k*_22_ become beneficial. Thus, severe trade-offs can have important consequences until the mutant grows moderately well on the nonpermissive host—as expected from the simple fact that once the mutant can maintain itself on the nonpermissive host, the permissive host becomes irrelevant. The effect of reducing *B*_1_ input to 10^6^ is slight but in the direction of increasing the negative consequences of a trade-off.

#### 3.1.4. Sequential bacterial presentation changes little

**Figure 4.**
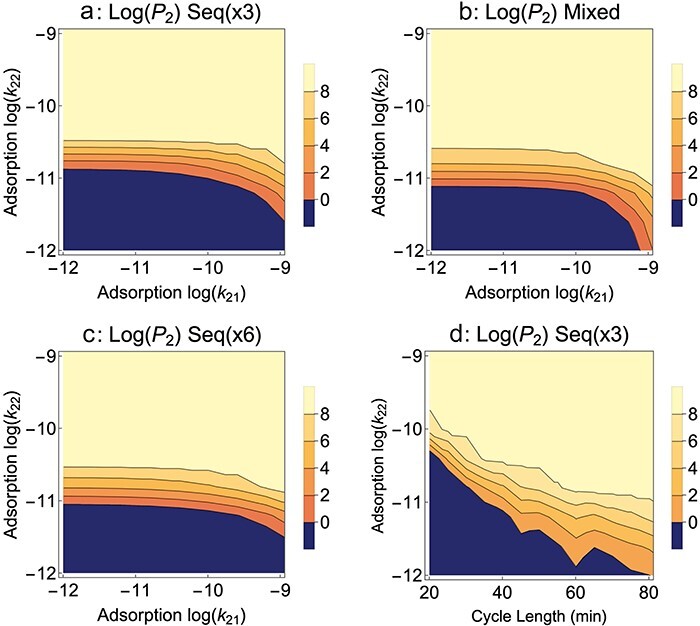
*B*_2_ blocks adsorption, Sequential presentation. Effects of Sequential presentation: alternating growth on pure *B*_1_ versus pure *B*_2_. (a) growth on *B*_1_ every third cycle. (b) Mixed presentation results from Fig. 3a are provided for comparison. There is little difference from Sequential in (a). (c) Growth on *B*_1_ every sixth cycle is slightly worse for mutant evolution than every third cycle, mirroring the effect of low *B*_1_ levels observed for Mixed presentation. (d) Effect of cycle length (*B*_1_ every third cycle). The same qualitative effects of exposure to *B*_1_ and cycle length are observed with Sequential presentation as with Mixed presentation. In (d), *P*_2_ levels are taken within a minute of cycle’s end once 580 min have elapsed; the contour jaggedness stems from the boosting effect of growth on *B*_1_ versus *B*_2_ and that the timing of *B*_1_ cycles relative to *P*_2_ sampling varies with cycle length. Parameter values are as given in Fig. 1; *k*_12_ = 0.

An alternative to mixing hosts in a single culture is to alternate hosts (Sequential presentation). Then, instead of deciding how much of each host to add to a single culture, one must decide how often to expose the phage population to a culture of *B*_1_ versus a culture of *B*_2_. Other design properties that were considered for Mixed presentation must also be addressed for Sequential: cycle length and dilution. Results from a Sequential protocol designed to mimic a favorable Mixed protocol are shown in Fig. 4a and compared to the corresponding Mixed protocol results in Fig. 4b: they are effectively indistinguishable. It is possible to qualitatively reclaim most of the results of Mixed presentation under different protocol variations: Fig. 4c shows that less-frequent growth on *B*_1_ is somewhat detrimental to mutant advance, and Fig. 4d shows that longer cycles are better when adsorption rates on *B*_2_ are low.

The near equivalence of Mixed and Sequential presentations for the evolution of host range shown here is not necessarily obvious. Efforts to model the evolution of generalists versus specialists—which is the ecological equivalent to the problem here—often find different outcomes for temporal heterogeneity than for temporal and spatial homogeneity (Kassen 2002, Bono et al. 2017). However, the protocols here do not mimic natural environments (as assumed in standard evolutionary models) because hosts are periodically replenished in the protocols. This replenishment changes the nature of competition—as indicated by the “coexistence” of both phages in some of the “Mixed presentation” parameter space of Fig. 1; predictions from standard theory preclude coexistence in homogeneous environments under the conditions of Fig. 1. These results also contrast with the clear superiority of Sequential over Mixed presentation in models of phages evolved to infect multiple hosts (Bull et al. 2022). However, those protocols differed from the protocols here in that there was no common host for all phages in the previous models. Overall, the lack of generality across these different studies testifies to the need to model protocol specifics and to develop intuition specific to those protocols.

#### 3.1.5. Other variations

We have explored two other variations of protocol: a continuous flow system (chemostat) in which uninfected bacteria are input at a constant rate and all contents flow out at the same rate, and a protocol similar to “Appelmans” in which phages are grown on each host separately and then pooled before they are again grown on each host separately (Burrowes et al. 2019, Bull et al. 2022). Under initial conditions corresponding to those of Fig. 1a and b, the same broad pattern emerges in which high *B*_1_ input benefits mutant evolution at the lowest adsorption rates (Supplementary S1).

### 3.2. Model 2: evolution of a “virulent” temperate phage

Temperate phages are phages with two alternative forms of infection (Stewart and Levin 1984, Davidson et al. 1990, Hobbs and Abedon 2016, Howard-Varona et al. 2017). Some infections are lytic, killing the cell in minutes. But other infections follow a benign strategy of inserting the phage genome into the host genome (becoming a “prophage”) and residing there indefinitely and seemingly without detriment to the host. These prophage-carrying bacteria are known as lysogens. Either spontaneously or in response to environmental triggers, a lysogen’s prophage can initiate the lytic pathway that produces phage progeny (a process known as induction), but the spontaneous rate of induction is typically so low that there is no noticeable difference between the growth rate of the lysogen and that of the prophage-free host. For many temperate phages (e.g. lambda), lysogens can be superinfected by free phages that are the same as their prophage, but the lysogens are “immune” so that the superinfecting genome is destroyed without harming the lysogen or displacing the prophage.

The life history just described does not cover all forms of phages that can exist without killing their host. Some prophages may render their hosts resistant to superinfection; filamentous phages exist in a plasmid-like state inside their hosts, they render their hosts resistant to superinfection, but their progeny are secreted through the bacterial membranes without killing their host (Rakonjac et al. 2017). We limit our modeling to the temperate life history described in the preceding paragraph, as exemplified by phage lambda (Ptashne 2004, Casjens and Hendrix 2015).

Temperate phages are unsuitable for therapy because they can convert the bacterial pathogen into a lysogen that is then free to grow without being killed by the free temperate phages. Worse, many temperate phages carry virulence genes that are expressed from the lysogen and exacerbate the infection (Schroven et al. 2021). It might therefore seem that temperate phages are to be avoided for therapy, but they are sometimes the only kind of phage known for pathogens (Nale et al. 2016). (Furthermore, engineered temperate phages have been proposed for the alternate purpose of converting a bacterial genome from drug resistance to drug sensitivity (Edgar et al. 2012, Yosef et al. 2015), but this approach differs from therapy in which the phage is used to kill the bacterium.) Indeed, prophages are virtually ubiquitous in wild bacterial genomes and thus would offer a ready source of phages for those bacteria—if the temperate phages could be evolved to kill their own lysogens. There are even examples in which *vir* mutants of temperate phages have been isolated—*vir* mutants lytically infect and destroy their own lysogens and also fail to form lysogens. *Vir* mutants would be safe for therapy.

There is thus an obvious benefit of evolving *vir* mutants of temperate phages. But the problem is complicated because there are at least three traits differing between a temperate phage and its *vir* counterpart that are relevant to therapy: (i) the probability of lysogen formation when a temperate phage infects a naïve host, (ii) the spontaneous induction rate of the lysogen, triggering the lytic process, and (iii) escape from the lysogen’s immunity to create a lytic infection. From a therapeutic perspective, the goal is to reduce lysogen probability, increase the induction rate, and increase escape from immunity. But because of feedback effects in the temperate phage life cycle, selection could potentially favor changes in some traits that benefit therapy but simultaneously favor changes in other traits that work against therapy. Changes to one trait are likely to affect other traits (pleiotropic effects), so mutational effects on the different traits will often be correlated.

The existence of lysogens introduces a new protocol option: should the attempt to evolve a *vir* mutant include the addition of lysogens each cycle or just uninfected bacteria? A *vir* mutant grows on lysogens, so adding lysogens may speed the selection. But the lysogens will necessarily carry the wild-type phage genomes, so adding lysogens may work against *vir* evolution by diluting the mutant phage (because of their background induction rate). It is thus not clear whether to add lysogens or uninfected bacteria.

Our investigation of this problem will address the nature of selection on the three lysogeny traits and the relative gains from adding uninfected cells versus lysogens at each cycle. Protocol properties that were investigated for Model 1 should be generalized and thus are only minimally revisited.

#### 3.2.1. The model

Equations for Model 2 are given below. Where possible, notation follows the same rules as with Equation (1) and is given in Table 2. Again, the reader is assured that the paper can be understood without recourse to them. The notation given in Table 2 is useful for following some of the text.

**Table 2.** Notation for Model 2 (temperate phage).

Terms	Meaning	Values used (units)
Variables (functions of time)		
*B*_0_	Density of uninfected, susceptible bacterium	Initial values 10^8^ and 10^7^ (/ml)
*L_i_*	Lysogen of phage *i* (*i *= 1, 2)	Varies (/ml)
*P_i_*	Density of free phage *i* (*i *= 1, 2). *P*_1_ is the wild type, and *P*_2_ is the mutant	Initial value 10^8^ for *P*_1_; 1 for *P*_2_ (/ml), the latter at 8 min
*I_i_*	Density of bacteria undergoing lytic infection with phage *i* (*i* = 1, 2)	Varies (/ml)
Parameters		
*r*	Bacterial growth rate	0.02 (/min)
*k_ij_*	Adsorption rate constant of phage *i* on bacterium *j*: *i = *(1, 2), *j *= (0, 1, 2), where uninfected bacteria are distinct from the two lysogens	≤10^−9^ (ml/min)
*δ_i_*	Rate constant of transition from infected bacterium (*i*) to burst	0.05 (/min)
*b_i_*	Phage number of progeny from infection *i* (*i* = 1, 2)	*b*_1_ = *b*_2_ =50 (individuals)
*λ_i_*	Lysogeny probability of *P_i_* when infecting *B*_0_	Varies
*ι*_j_	Induction rate of lysogen *j* (*j* = 1, 2)	Varies
*σ_ij_*	Successful superinfection probability of phage *i* on lysogen *j; i*, *j *= (1, 2). These establish lytic infections	
*C* ^a^	Cycle length	20–90 (min)
Dilution^a^	Fraction of free phage transferred at cycle end	0.1

a These parameters are absent from Equation (2) but included in the code.

Equations for temperate phage evolution are different but not substantially more complicated than for Model 1. One simplification is that the model need only consider a single host bacterium (which can exist in a free state or as a lysogen). We also restrict the model to the wild type (*P*_1_) and one mutant phage (*P*_2_). There are, however, three new parameters: lysogeny probability (*λ*), induction rate (*ι*), and lytic superinfection probability (*σ*):


(2) $$\begin{array}{l} B_{0}^{^{\prime}} = {B_0}\left( {r - {k_{10}}{P_1} - {k_{20}}{P_2}} \right)\\ L_{1}^{^{\prime}} = {L_1}\left( {r - {\iota _1}} \right) + {\lambda _1}{k_{10}}{P_1}{B_0} - {\sigma _{11}}{k_{11}}{P_1}{L_1} - {\sigma _{21}}{k_{21}}{P_2}{L_1}\\ L_{2}^{^{\prime}} = {L_2}\left( {r - {\iota _2}} \right) + {\lambda _2}{k_{20}}{P_2}{B_0} - {\sigma _{22}}{k_{22}}{P_2}{L_2} - \,{\sigma _{12}}{k_{12}}{P_1}{L_2}\\ I_{1}^{^{\prime}} = \left( {1 - {\lambda _1}} \right){k_{10}}{P_1}{B_0} + {\iota _1}{L_1} - {\delta _1}{I_1} + {P_1}\left( {{\sigma _{11}}{k_{11}}{L_1} + {\sigma _{12}}{k_{12}}{L_2}} \right)\\ P_{1}^{^{\prime}} = {b_1}{\delta _1}{I_1} - {P_1}({k_{10}}{B_0} + {k_{11}}{L_1} + {k_{12}}{L_2})\\ I_{2}^{^{\prime}} = \left( {1 - {\lambda _2}} \right){k_{20}}{P_2}{B_0} + {\iota _2}{L_2} - {\delta _2}{I_2} + {P_2}\left( {{\sigma _{21}}{k_{21}}{L_1} + {\sigma _{22}}{k_{22}}{L_2}} \right)\\ P_{2}^{^{\prime}} = {b_2}{\delta _2}{I_2} - {P_2}\left( {{k_{20}}{B_0} + \,{k_{21}}{L_1} + {k_{22}}{L_2}} \right) \end{array}$$

#### 3.2.2. Evolution of lysogeny probability (λ_2_)

As one of the first steps after infection of a naïve host, a temperate phage adopts either a lytic or lysogenic pathway. The lysogenic pathway involves the expression of an integrase gene to insert the phage genome into the host genome. Engineered deletions of integrase have been used to render a temperate phage suitable for treatment (Dedrick et al. 2019), so here we ask whether it is practical to direct the evolution of reduced lysogeny without engineering—whether nonintegrating mutant phages will evolve and under what conditions.

**Figure 5.**
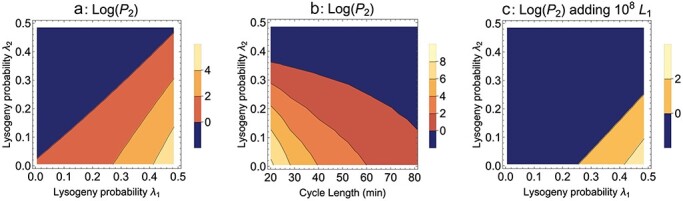
Temperate phage, Mixed presentation. Evolution of lysogeny probability (*λ*_2_ for the mutant, *λ*_1_ for the wild type). (a) A lower probability is favored under serial transfer, the more so the greater the difference from the wild type. (b) Evolution of lysogeny probability per cycle length. There is a moderately strong benefit of short cycle lengths, consistent with selection for rapid growth and limited time for lysogens to form. (c) Evolution of a lower lysogeny rate is hindered by adding 10^8^ wild-type lysogen each cycle (*L*_1_ is the wild-type lysogen). Bacterial and lysogen growth rate = 0.02/min, cycle length = 50 min, dilution = 1/10, all burst sizes = 50, all transition rates from the infected state to burst = 0.05, adsorption rates of both phages to all hosts = 10^−9^ ml/min. Induction rates of lysogens = 0.003/min, lysogen probability of the wild type = 0.4, lytic superinfection probability = 0.01. Per cycle input of the permissive host (*B*_0_) is 10^8^; initial value of the wild-type phage = 10^8^ and that of the mutant phage = 1 at 8 min.

Intuition suggests that reduced lysogeny (hence a higher fraction of infections immediately become lytic) will increase phage fitness when there is an abundance of uninfected hosts. An abundance of hosts selects for fast growth—especially for a short generation time (Bull 2006, Bull et al. 2011). Under these conditions, entering the lytic pathway should be favored because it results in a faster generation of progeny than when entering the lysogenic pathway (the burst size should not be affected). This expectation is borne out (Fig. 5a). The mutant ascends faster the more it avoids lysogeny relative to the wild type. Importantly, the protocol should use conditions under which the wild type has a high rate of lysogen formation; if the wild-type rate is low, then selection is weakened. Thus, the best protocol provides an abundance of uninfected hosts under conditions for which the wild type forms lysogens; this balance may not be easily achieved.

Figure 5b shows that shorter cycles enhance the mutant phage’s advantage when it has a lower lysogeny probability. The benefit of shorter cycles is presumably that there is less time for lysogens to accumulate and less time for uninfected hosts to be cleared. In view of results for Model 1, it is interesting and perhaps surprising that shorter cycles have such an advantage despite the greater number of dilutions. The effect of cycle length here is opposite that observed for Model 1 (adsorption block) and highlights the sensitivity of the best protocol to the nature of the growth block.

Adding the same number of lysogens every cycle as uninfected bacteria (10^8^) is substantially detrimental to mutant evolution (Fig. 5c versus Fig. 5a). From intuition, the addition of lysogens is not expected to benefit selection for reduced lysogen formation, but it is not necessarily clear that doing so would greatly impede selection, either.

#### 3.2.3. Evolution of induction rate (ι_2_)

**Figure 6.**
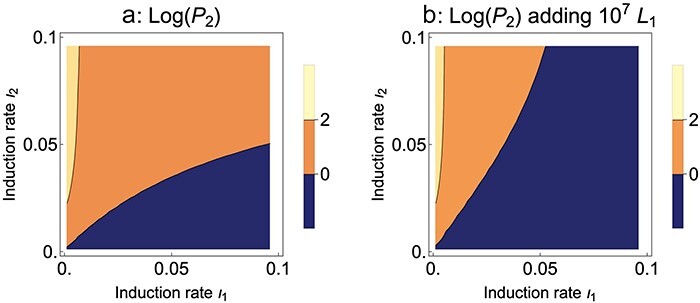
Temperate phage, Mixed presentation. Evolution of induction rate (*ι*_2_ for the mutant, *ι*_1_ for the wild type). (a) An increase in induction is favored under serial transfer, but selection is weak unless the wild-type induction rate is very low and the mutant rate is substantially higher. (b) Adding 10^7^ wild-type lysogens per cycle weakens selection of the mutant (there are no useful contours when adding 10^8^). Parameter values are as given in Fig. 5

Spontaneous induction initiates the lytic pathway in a lysogen. Thus, a high induction rate parallels a failure to form lysogens: as the induction rate gets high, lysogeny becomes ever shorter, and in the extreme, there is no lysogeny. Figure 6a shows that a higher induction rate is indeed favored during serial transfer. However, the evolution is rapid only if the wild-type induction rate is very low (<0.01), and even then, the evolution is not as rapid as with the most rapid evolution of lysogeny avoidance. Adding (10^7^) wild-type lysogens to each cycle is highly counterproductive (Fig. 6b versus Fig. 6a).

#### 3.2.4. Evolution of lytic superinfection (σ_21_)

**Figure 7.**
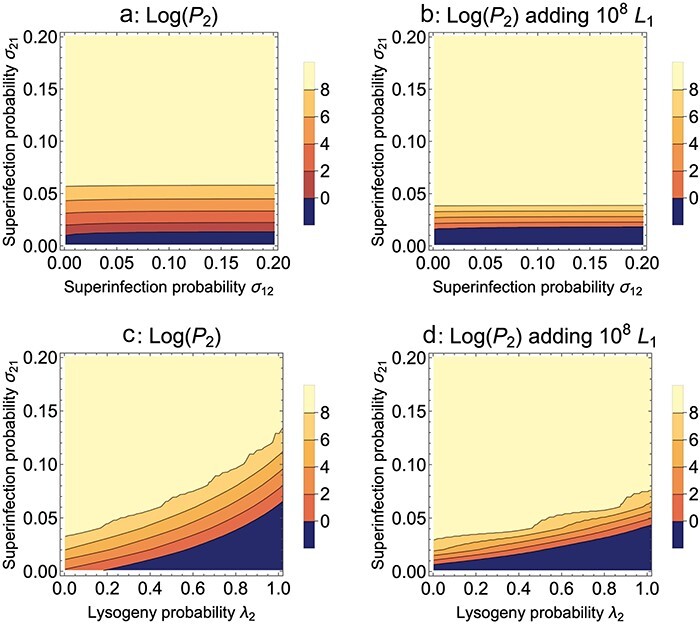
Temperate phage, Mixed presentation. Evolution of lytic superinfection: *σ*_12_ is for wild-type phage infecting a mutant lysogen, *σ*_21_ is for a mutant phage infecting a wild-type lysogen. (a) Lytic superinfection of a wild-type lysogen by the mutant begins to be favored once the probability of lytic superinfection exceeds ∼ 0.01. The evolution is insensitive to the probability of the wild-type phage lytically superinfecting a mutant lysogen. (b) Adding 10^8^ wild-type lysogens (*L*_1_) per cycle somewhat compresses the intermediate zone of mutant evolution, but there is a slightly increased threshold for the mutant to advance (the dark blue region extends slightly higher). (c) Mutant evolution is expedited when changing both lysogeny and lytic superinfection probabilities together. The effects combined are as expected from their separate effects. Recall that lower values of *λ*_2_ are favored. (d) Adding wild-type lysogens improves joint selection of lysogeny and lytic superinfection probability in some zones but worsens it in others—note the no-evolution zone (dark blue) extends further to the left in (d) than in (c). Parameter values are otherwise as given in Fig. 5

The most desirable outcome is avoiding death of the superinfecting phage upon infection of a lysogen. Mechanistically, this ability would be gained by the mutant’s escape from wild-type immunity—in phage lambda by its operator sequences no longer binding the wild type’s repressors. Figure 7 shows that lytic superinfection by the mutant is strongly favored when its probability exceeds ∼0.05, but the mutant ascends at least somewhat when the probability exceeds 0.01. In these trials, the mutant has the same probability of lysogeny and induction as does the wild type, so the only variable is lytic superinfection. This evolution is effectively independent of the wild type’s lytic superinfection probability on lysogens (*σ*_12_). Trials in which the mutant initiated lytic superinfection with the same probability on wild-type lysogens as on mutant lysogens (*σ*_21_ = *σ*_22_) showed essentially no effect of *σ*_22_ (not illustrated).

In contrast to the evolution of lysogen formation and induction rate, the evolution of lytic superinfection is somewhat enhanced by the addition of lysogens in some respects but not others: the threshold for evolution of the mutant in the highest contour expanded slightly, but the blue zone of no evolution also expanded somewhat (compare Fig. 7a to Fig. 7b). Thus, for some zones, the drawback of adding wild-type lysogens (which boosts the abundance of wild-type phage through induction) is more than offset by providing a host on which the mutant has a big enough advantage.

Since the evolution of mutant lytic superinfection probability *σ*_21_ is largely independent of *σ*_22_ and *σ*_12_, we can ask about the joint evolution of lytic superinfection and lysogeny probabilities (Fig. 7c and d). Mutants that change both parameters in the individually favored directions have an even greater advantage than with either change alone. Furthermore, the addition of wild-type lysogens (*λ*_1_) enhances the evolution in some parts of the space but worsens it in other parts (Fig. 7d).

The models suggest that the evolution of temperate phages to benefit therapy is practical. A single protocol selects all three traits in the desired directions: reduced lysogeny probability, higher induction, and higher lytic superinfection. The protocol is simply one of exposing the phage population to uninfected bacteria each cycle; the addition of lysogens at the start of each culture is counterproductive to some of this selection but not all, and it never seems to offer much benefit.

### 3.3. Model 3: the nonpermissive host aborts infection

Bacteria use several mechanisms to abort phage infections (Durmaz et al. 1992, Labrie et al. 2010). Some kill the host while killing the phage; others allow the host to survive. Independently of host survival, some abortive infection mechanisms do not coevolve with the phage; others (especially CRISPR) evolve blocks as the phage evolves to overcome them (Levin et al. 2013, Rodríguez-Román et al. 2024). Here, we assume the simple case of a fixed block and that the abortive host dies when it is infected by the wild-type phage.

When the nonpermissive block is due to an abortive infection, the selection of an escape mutant becomes complicated. Starting from a wild type that adsorbs well to the nonpermissive host (and dies upon infection), there is a selection for mutants that avoid the nonpermissive host but continue to infect the permissive host (Bull 2006, Heineman et al. 2008). These avoidance mutants are not only useless for killing the nonpermissive host but are counterproductive to the attempted selection: they are not easily detected by plating because they plaque poorly or not at all on the nonpermissive host, but in culture, they will displace the wild type and interfere with the evolution of other kinds of mutants. As explained later, the path of evolution will depend on the order of different classes of mutations and on protocol details such as cycle length. We develop the models in sequence: (I) evolution of an avoidance mutant on a background of the wild type, (II) evolution of a growth mutant on a background of the wild type, and then (III) evolution of a growth mutant on a background of avoidance. All processes assume Mixed presentation.

#### 3.3.1. The model

Equations for Model 3 are given below. Where possible, notation follows the same rules as with Equation (1) and is given in Table 3. We reserve *P*_2_ for the mutant that grows on the nonpermissive host (*B*_2_) to maintain consistency with Model 1; *P*_3_ is used for the phage that avoids the abortive host. Terms are omitted for infections that produce no phage progeny:


(3) $$\begin{array}{l} B_{1}^{^{\prime}} = {B_1}\left( {r - {k_{11}}{P_1} - {k_{21}}{P_2} - {k_{31}}{P_3}} \right)\\ B_{2}^{^{\prime}} = {B_2}\left( {r - {k_{12}}{P_1} - {k_{22}}{P_2} - {k_{32}}{P_3}} \right)\\ P_{1}^{^{\prime}} = {b_{11}}{\delta _{11}}{I_{11}} - {k_{11}}{P_1}{B_1} - {k_{12}}{P_1}{B_2}\\ I_{11}^{^{\prime}} = {k_{11}}{P_1}{B_1} - {\delta _{11}}{I_{11}}\\ I_{12}^{^{\prime}} = {k_{12}}{P_1}{B_2} - {\delta _{12}}{I_{12}}\\ P_2^{^{\prime}} = {b_{22}}{\delta _{22}}{I_{22}} + b_{21} \delta_{21} I_{21}-{k_{21}}{P_2}{B_1} - {k_{22}}{P_2}{B_2}\\ I_{21}^{^{\prime}} = {k_{21}}{P_2}{B_1} - {\delta _{21}}{I_{21}}\\ I_{22}^{^{\prime}} = {k_{22}}{P_2}{B_2} - {\delta _{22}}{I_{22}}\\ P_3^{^{\prime}} = {b_{31}}{\delta _{31}}{I_{31}} - {k_{31}}{P_3}{B_1} - {k_{32}}{P_3}{B_2}\\ I_{31}^{^{\prime}} = {k_{31}}{P_3}{B_1} - {\delta _{31}}{I_{31}} \end{array}$$

**Table 3.** Notation for Model 3 (abortive infection).

Terms	Meaning	Values used (units)
Variables (functions of time)		
*B_i_*	Uninfected density of bacterium *i* (*i *= 1, 2). *B*_1_ is permissive, and *B*_2_ nonpermissive	Initial values 10^8^ and 10^7^ (/ml)
*P_i_*	Density of free phage *i* (*i *= 1, 2, 3). *P*_1_ is the wild type, *P*_2_ is the mutant with a small burst on *B*_2_, and *P*_3_ is the avoidance mutant with zero burst if it infects *B*_2_	Initial value 10^8^ for *P*_1_; 1 for *P*_2_ and *P*_3_ (/ml), the latter at 8 min
*I_ij_*	Density of “infected” bacterium *ij* (phage *i* in bacterium *j* (*i* = 1, 2, 3; *j* = 1,2)	Varies (/ml)
Parameters		
*r*	Bacterial growth rate	0.02 (/min)
*k_ij_*	Adsorption rate constant of phage *i* on bacterium *j*	≤10^−9^ (ml/min)
*δ_ij_*	Rate constant of transition from infected bacterium (*ij*) to burst	0.05 (/min)
*b_ij_*	Phage number of progeny from infection *ij*	*b*_11_ = *b*_21_ = *b*_31_ = 50; *b*_22_ = 2; *b*_12_* = b*_32_* = *0 (individuals)
*C* ^a^	Cycle length	20–90 (min)
Dilution^a^	Fraction of free phage transferred at cycle end	0.1

a These parameters are absent from Equation (3) but included in the code.

#### 3.3.2. Evolution of avoidance

An abortive host was used to select phage T7 variants that avoided that host but continued to grow on other strains (Heineman et al. 2008). Using hosts *Escherichia coli* C, B, and K_12_, T7 was selected to avoid the K12 host or to avoid the B host while maintaining growth on C. Both selections resulted in an ∼10-fold decrease in adsorption to the abortive host while maintaining growth on the other two hosts, but adsorption to the other hosts was affected. Although the abortive host used in that study (a deletion of the host gene encoding thioredoxin) was known to be insurmountable by T7—and thus host avoidance was the only evolutionary path available to the phage—the study shows the potential to rapidly select avoidance when mutants capable of growth are rare. We thus start with evolution of avoidance.

**Figure 8.**
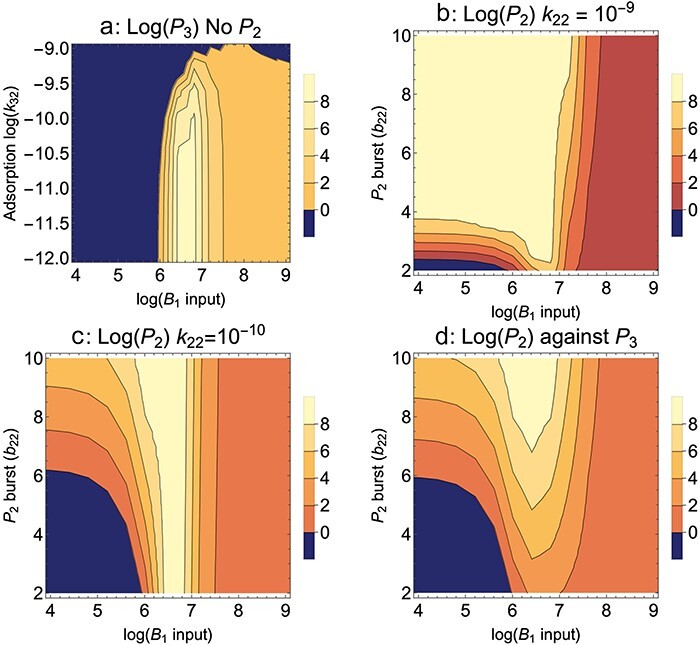
Abortive host, Mixed presentation. Evolution is sensitive to the different kinds of mutants that arise. *P*_1_ is the wild-type phage that is killed/aborted by *B*_2_; *P*_2_ is a mutant phage that produces a small burst on *B*_2_; *P*_3_ is a mutant phage that somewhat avoids *B*_2_ but is killed by *B*_2_ when it infects. (a) Evolution of an avoidance mutant (*P*_3_) in a population of wild-type phage (*P*_1_); the mutant has reduced adsorption to the abortive host. The fate of the avoidance mutant depends heavily on input of the permissive host (*B*_1_) and only modestly on the adsorption rate once the adsorption rate drops below ∼10^−9.5^. Partial avoidance instead of complete avoidance is thus a likely empirical outcome if it arises. (b) Evolution of a partial-growth mutant (*P*_2_) in a population of wild-type phage (*P*_1_); phage *P*_3_ is absent. Evolution of the mutant depends on burst size and permissive host input, *B*_1_. For a burst size of <4 on the abortive host, the mutant fate rests heavily on specific levels of the permissive host up to 10^7^. Here, the mutant adsorption rate on the abortive host is the same as for the wild type, *k*_22_ =10^−9^. (c) The same as for (b), but the mutant adsorption rate on the abortive host is reduced to 10^−10^. The zone of mutant evolution is greatly narrowed. (d) Evolution of a low-burst mutant (*P*_2_) in a population of the avoidance mutant (*P*_3_); both adsorption rates on the abortive host are the same, 10^−10^, but the pattern is qualitatively the same if *k*_32_ =10^−11^. This case represents a later introduction of the partial-growth mutant (*P*_2_) compared to that of the avoidance mutant (*P*_3_), so the avoidance mutant has already swept. Parameter values are as given in Fig. 1 (parameter values are the same regardless of subscript), except that *k*_12_ = 10^−9^ and *b*_12_ = 0. Initial values are 10^8^ for the common phage, 1 for the invading phage at 8 min.

When the avoidance mutant is the only one competing with the wild type, it easily evolves provided *B*_1_ levels are sufficiently high (Fig. 8a). Note that we consider a range of low adsorption rates, not just complete avoidance. (*P*_3_ is designated as the avoidance mutant, so *k*_32_ is its adsorption rate on the abortive host.) The unexpected results are (i) an extreme sensitivity of the 10 h density of *P*_3_ to permissive host levels (*B*_1_) and (ii) a relative insensitivity to the level of avoidance (adsorption rate) below ∼10^−9.5^. In hindsight, these results can be justified. At low *B*_1_, phages are lost because they are killed off too quickly by adsorption to the abortive host. At high *B*_1_ levels, the mutant is slow to ascend because of competition with wild-type phage—which is maintained at high levels by the abundant permissive host. The relative insensitivity of evolution to the adsorption rate at small values is due to large effects from small changes in the exponent. The change from 10^−9.0^ to 10^−9.5^ seems small but results in approximately twice as many phages surviving for 10 min (with a cell density of 10^8^); in 10 h, this becomes a large effect. Even greater drops in adsorption will hasten this evolution but cannot yield much higher final densities because of the limits imposed by dilution every cycle.

#### 3.3.3. Evolution of a small burst

Evolution of a mutant phage (*P*_2_) with a small burst on the nonpermissive host, when evolving against a wild-type phage (*P*_1_), is also sensitive to *B*_1_ levels (Fig. 8b and c). When the adsorption rate on *B*_2_ is high (*k*_22_ = 10^−9^), this sensitivity decreases for larger bursts at low B_1_ input, but the sensitivity remains when the adsorption rate is low (*k*_22_ = 10^−10^).

Suppose that the first mutant to ascend is an avoidance mutant, then a second mutant phage arises, one that has a small burst on the nonpermissive host. This second mutant would seem to have an advantage over both the wild type and the avoidance mutant, but such is only partly true. It will indeed have an advantage over the wild type. But it lacks an advantage over the avoidance mutant early in each cycle because infecting the “poor” host reduces its growth rate until only the nonpermissive host remains (Bull 2006, Heineman et al. 2008). Thus, when we consider the evolution of a small-burst mutant (*P*_2_) after the avoidance mutant (*P*_3_) has swept the population and displaced the wild type (Fig. 8d), the evolution of the low-burst mutant is now substantially delayed compared to its evolution against just the wild type.

These limited trials show that protocol has a substantial impact on the directed evolution of growth on the nonpermissive, abortive host. They also show that two different outcomes are possible, only one of which leads to actual growth on the new host. From the analyses shown and from others we have conducted, there are few suggestions of generalities other than to be wary of evolving avoidance mutants—the protocol variations likely to matter most may be specific to the phage and bacteria and thus will need to be evaluated on a case-by-case basis.

## 4. Discussion

With the ongoing revival of phage therapy—the use of bacteriophages to treat an infection—there are occasions in which phages are lacking for treating a specific bacterium (Schooley et al. 2017, Dedrick et al. 2019, 2021, 2023, Pirnay et al. 2024). Three approaches to acquiring a phage for a particular bacterium are evident: screening samples from the environment, using directed evolution of an existing phage to obtain a mutant that grows on the new host, and engineering a phage to modify its host range. Here, we study the second method.

Adapting a phage to grow on a new host is classically done by plating and screening for plaques on that host. The ability to grow and plate large numbers of bacteria means that this kind of screening can be successful when plaque-forming mutants are as rare as possibly 10^−10^, as it is often feasible to plate 10^10^ phage. Screening for plaques is the obvious first step in attempting to evolve a phage onto a new host. But a plaque-forming mutant is a large-effect change. When only small-effect mutations are available, a protocol may be required that accumulates those mutations without direct evidence that growth on the new host is improving, at least until several mutations have ascended in the phage population that collectively yield tangible evidence. There are various ways such protocols might be implemented, and it is desirable to know in advance which variations are most likely to foster success. Here, we have provided computational analysis of mathematical models to assess the outcomes from different protocol variations for three different types of blocks to phage infection: an adsorption block, a temperate phage unable to grow on its own lysogen, and an abortive infection. These models assume a growth environment of mass action, as would be appropriate for liquid culture, and serial transfer across multiple cultures—since any single culture will not be grown long enough to allow a small-effect mutant to accumulate to high frequency. They all involve some phage growth on the permissive host along with growth on the nonpermissive host. They all assume that the mutant is initially present at low frequency, such that the effect of the protocol on the selection of the mutant can be evaluated.

Our analyses suggested that the most obvious protocol variations for evolving to overcome an “adsorption block” are approximately equivalent. Thus, it does not matter whether the permissive and nonpermissive hosts are mixed in a single culture or presented to the phage pool sequentially. There is a modest detrimental effect of growing the phage pool through many short-term cultures as opposed to fewer long-term cultures because each transfer of a culture involves a dilution that sets back phage numbers. The main surprise was that including large numbers of the permissive host along with the nonpermissive host can be beneficial but only if the wild-type phage virtually failed to adsorb to the nonpermissive host. Our models did not allow bacterial densities to enter a realm in which bacterial growth would slow due to crowding and nutrient exhaustion; however, such considerations could apply at high bacterial densities.

Evolving a “temperate phage” to acquire any of the three “virulent” properties appears to be relatively straightforward (reduced lysogen formation, a higher spontaneous induction rate, and a higher probability of lytically superinfecting lysogens). Thus, a single protocol of serial transfer on uninfected bacteria favors desirable changes in all three properties, although the selection is weak for the induction rate. Adding lysogens to the culture somewhat improves the evolution of lytic superinfection, but the effect is small and works against the evolution of rapid induction. Evolution of reduced lysogen formation benefits from a protocol in which the wild-type phage has an intrinsically high rate of forming lysogens on infection.

In contrast, gradually evolving a phage to grow on the “abortive host” presents several challenges. The biggest problem is that the attempt to evolve growth is likely to favor nongrowth—to favor a mutant that avoids the abortive host altogether while maintaining itself on just the permissive host. Furthermore, the evolution of a mutant that has a small burst on the otherwise abortive host is highly sensitive to the density of permissive hosts added to the cultures. We have little constructive advice to offer on best protocols. If plaque mutants cannot be obtained, the simplest solution may be to screen environmental phage isolates or to use engineering.

The problem addressed here is different from the evolution of broad host range phages (Mapes et al. 2016, Burrowes et al. 2019, Bull et al. 2022). The selection for growth on a new host considered here may increase host range or keep it the same (if growth on the new host results in a loss of growth on the previous host). Protocols for evolving broad host ranges have been considered separately and commonly use the Appelmans protocol (Mapes et al. 2016, Burrowes et al. 2019, Vu et al. 2024).

Genetic engineering of phages might seem to supplant any need to apply lengthy protocols for the selection of small-effect mutations. However, genetic engineering itself does not ensure the attainment of large-effect mutations capable of detection by plaque assays, and if only small-effect mutations are available through engineering, the protocols here will be necessary. Even when engineering might possibly yield such ideal mutants, its success requires extensive infrastructure specific to individual phages, requires prior knowledge of genes likely to be involved in host range, and may face regulatory constraints that do not apply to naturally evolved phages. Furthermore, the empirical evolution of a change in host range of a phage is potentially useful for informing efforts to engineer phages. Engineering methods for expanding phage host range necessarily target specific phage genes, such as tail components (Dedrick et al. 2019, Yehl et al. 2019, Latka et al. 2021). The selection of host range changes, as modeled here, will reveal whatever mutations nature provides. Thus, a directed evolution approach can lead to discovery of unanticipated mechanisms of host range evolution (such as a chaperone; Hashemolhosseini et al. 1994) that could be exploited by engineering.

### 4.1 *In vitro* to *in vivo*

The goal here has been to identify “best” lab protocols for evolving phages to use in therapy. It is obvious that some aspects of phage performance *in vitro* may not match *in vivo*, given the many differences between media and tissue fluids. Are the results of our study therefore suspect? We suggest not. Our study has been developed for the directed evolution of qualitative traits that are likely to transcend differences between *in vitro* and *in vivo*: host range, loss of temperate behavior, and overcoming abortive infections. With respect to host range, phage therapy relies on the *in vitro* screening of phages for use inside a host. If host range was often sensitive to the environment, practitioners of phage therapy could not routinely use *in vitro* screening to identify useful phages, and a fundamentally different approach to phage screening would have been developed. Consequently, the evolution of a new host range *in vitro* should not be undermined *in vivo*. Likewise, the ability of a *vir* mutant of a temperate phage to grow on its own lysogens is a phage × phage interaction of genetics, not environment; a phage overcoming an abortive infection is also a property of phage overcoming host genetics operating inside the bacterium. We are thus confident that success *in vitro* will translate to success *in vivo*. We do, however, caution that many other properties of phage performance that evolve during *in vitro* adaptation (during “phage training”) may be sensitive to the environment of adaptation, especially the quantitative values of adsorption rates, as they could well be affected by differences between media and tissue fluids.

### 4.2 Empirical tests

The results here derive entirely from modeling. How might they be useful to empiricists? Perhaps the biggest challenge in evolving phage host range is obtaining the relevant mutations. In many cases, mutations allowing growth on the nonpermissive host may be inaccessible in any feasible population size. For example, phage T7 (and many other phages) have an absolute requirement of the host factor thioredoxin; T7 has never been observed to evolve growth on hosts that are null for this product (Qimron et al. 2006), but T7 phages engineered to carry the host gene can grow on those hosts (Bull et al. 2024). No worldly feasible population of phages would ever carry a natural mutation by which a wild-type T7 acquired a complete gene for thioredoxin, so this is effectively an insurmountable block to all natural evolutionary methods. Mutation supply will confront any attempt to evolve phage host range, and although steps may be taken to enhance the mutation supply—use of large phage populations, mutagenesis, recombination among related phages, and even engineered, random phage libraries—not all efforts will succeed. There is yet little predictive ability of whether an uncharacterized phage will experience the mutations enabling it to grow on a new host.

Mutation supply lies outside the bounds of the present study. Instead, we considered how the protocol affects the ascent of mutations that do enable growth. The first distinction is whether single mutations allow “visible” growth on the new, nonpermissive host. If they exist, then no special protocol is required. If only small-effect mutations are available, then the protocols here apply. The protocol variations analyzed here could be tested directly, by comparing rates of adaptation to the new host under the different conditions of dilution, cycle length, and so on. But the more practical use of the work here is to enhance awareness of protocol features whose benefits are self-evident in hindsight. For example, when attempting to evolve adsorption to a new host, minimal dilution and long exposure before transfer have easily understood benefits. Even more basic, the realization that adaptation to a new host may occur in small steps may prove useful when large-effect mutations are not evident. Likewise, the realization that *vir* mutants of temperate phage may be best evolved on nonlysogens (and how to encourage that evolution) may prove useful without prior tests of the model variations we considered. The challenges associated with evolving a phage to overcome an abortive infection might lead the practitioner to nonevolutionary approaches, such as environmental screening. We view these kinds of insights to provide the main utility of this study, without the need for formal tests of each protocol variation.

## 5. Conclusions

Phages can often be evolved to grow on new host bacterial strains that initially do not support phage growth. Evolving a phage to grow on a new host is not always trivial, as evidenced by efforts to engineer phage libraries that enhance variation for host range expansion (see references earlier). Here, we have used computational models (of ordinary differential equations) to address one dimension underlying the evolution of phage host range: the effect of protocol on ascent of small-effect mutations enabling growth on the new host. All models assumed serial transfer in liquid culture, which is perhaps the most practical empirical environment for laboratory evolution. We considered three different types of block to phage infection: adsorption, temperate phage immunity, and abortive infection. The overall finding is that no single protocol is best for the different types of block. For example, long times between transfer are best when evolving to overcome a block to adsorption but not when evolving a *vir* mutant of a temperate phage. At least in hindsight, many of the findings can be justified intuitively. We suggest that the main benefit of the work is to provide practical insights for the empiricist, and the intuitive nature of the results means that many of the findings can be applied prior to empirical testing. Limited modeling may be advisable in advance of protocols that deviate from those analyzed here.

## Supplementary Material

veae100_Supp

## Data Availability

All data used in this study may be generated by the Mathematica files provided in the Supplementary material.
