# Supplementary material for: Mathematical comparison of protocols for adapting a bacteriophage to a new host
Source: Virus Evol. 2024 Nov 22;10(1):veae100. doi: 10.1093/ve/veae100 (PMC11665826; doi:10.1093/ve/veae100)
Supplement: veae100_Supp [file veae100_supp.zip › veae100_Supp/Supplement S1.pdf]

Supplement S1: Modeling alternatives

Supplement to: Mathematical Comparison of Protocols for Adapting a Bacteriophage to a New Host

By JJ Bull, SM Krone

I. Robustness of phage evolution to different models of lysis timing

Various methods for modeling phage growth have been proposed. The biology of the process is well established for lytic phages in liquid media: infection is followed by phage replication and assembly inside the cell, which results in burst of the infected cell and release of phage progeny (Adams 1959). Once cell density is specified, phage reproduction can be reduced to three easily estimated parameters: an infection rate, a time from infection to lysis, and a burst size. Infection is usually modeled as a mass action process, as the product of phage density, cell density, and an adsorption rate constant that is on the order of 10^{-8} – 10^{-12} mL/min (Adams 1959).

Although the three components of phage reproduction have received extensive empirical study over the last half century, there has been less attention to testing predictions of phage growth as a function of those components. Perhaps the most extensive attempt to match actual phage growth rate with model predictions is that in (Bull, Heineman and Wilke 2011) applied to phage T7. The culture system was one of serial transfer while maintaining an excess of cells, with separate estimates of adsorption rate, lysis time, and burst size. Observed T7 growth rates were in excess of 40 doublings/hr (just over a 10^{12}-fold increase per hour). The model fit to the data yielded a slightly lower growth rate than observed, but predicted fitness was highly sensitive to slight variations in some parameters (especially lysis time) that could not be resolved empirically.

A major emphasis of that study was the effect of culture asynchrony on fitness estimations. One of the consequences of a narrow distribution of lysis times (e.g., of a fixed lysis time) is that cultures remain highly synchronous soon after and potentially long after phage are first added. If the adsorption rate constant is high, most phages will infect cells within a few minutes of their addition to the culture. Then, with a 30 minute lysis time (for example), the culture will consist of infected cells but few free phage over the next ~25 minutes. Then the bursts of separately infected cells will also be largely synchronous, and the synchrony propagates with the next round of infections. The synchrony will decay over time, eventually attaining a 'stable-age-of-infection' distribution. At that point, the proportions of free phage and of different stages of infection remains constant. Once the infection states reach a steady state, and assuming an ongoing excess of cells, phage growth rate is determined strictly by cell density, the adsorption rate constant, burst size, and the distribution of lysis times (Bull 2006; Bull, Heineman and Wilke 2011).

For accurately modeling phage growth rate in an excess of cells, correctly modeling the distribution of lysis times is critical. However, when comparing the relative fitnesses of different phages that vary in burst size or adsorption rate, the importance of accurately modeling lysis time is not clear. For example, when two phages are modeled with the same lysis time properties, fitness effects of burst size or adsorption rate differences may transcend lysis time assumptions. The models we have used for this study impart a biologically unrealistic

distribution of lysis times. The goal of this Supplement is to determine whether our conclusions are likely to be affected in important ways.

Using ordinary differential equations, the time to lysis has been modeled in at least four different ways:

i) A fixed time to lysis, using delay-differential equations (Campbell 1961)
ii) A distribution of lysis times centered on the average (Bull, Heineman and Wilke 2011)
iii) A single lysis rate parameter, resulting in an exponential distribution of lysis times (Weitz 2016)
iv) Immediate lysis (Levin and Bull 1996)

Although (i) and (ii) can be solved for equilibrium conditions (Campbell 1961; Levin, Stewart and Chao 1977), they can present computational challenges for analyses of extensive time courses. Furthermore, because the protocols analyzed here involve periodic dilutions of phage densities, any approach using stored, past densities faces additional problems in ensuring that past densities are reduced according to the dilution. A process assuming a fixed time to lysis can be encoded directly in discrete-time programs written specifically for the application (e.g., Bull, Heineman and Wilke 2011) but is tedious. The advantages of (iii), which we adopt, is that it smooths dynamics and simplifies the numerical analyses while allowing specification of an average lysis time [the approach in (iv) does not allow for an average lysis time]. However, because an exponential lysis time is biologically unrealistic, it is important to determine that this assumption does not lead to erroneous results.

In Fig. S1, we compare the effects of an exponentially distributed lysis time (panels A and B) versus a gamma distributed lysis time (C and D) versus a directly encoded fixed lysis time (E and F), all for the case of text Fig. 1. For the gamma, a 12-step transition was modeled in which all rates were identical (resulting in a gamma distribution with a shape parameter of 12); the mean lysis time was maintained the same as for the exponential distribution at 20 mins. This gamma has a mode of 18.3, close to the mean, and a low density at early times – profoundly different from an exponential. For the directly encoded fixed lysis time, a C code program was written with a step size of 0.01 minutes. All methods used the same parameterization as in Fig. 1: 20 minute average lysis time, 50 minute cycles, burst size 50 and 10^{-9} mL/min adsorption rates to B_1.

The three sets of contour plots show quantitative differences but maintain the same broad features as with the exponential distribution in (A) and (B).

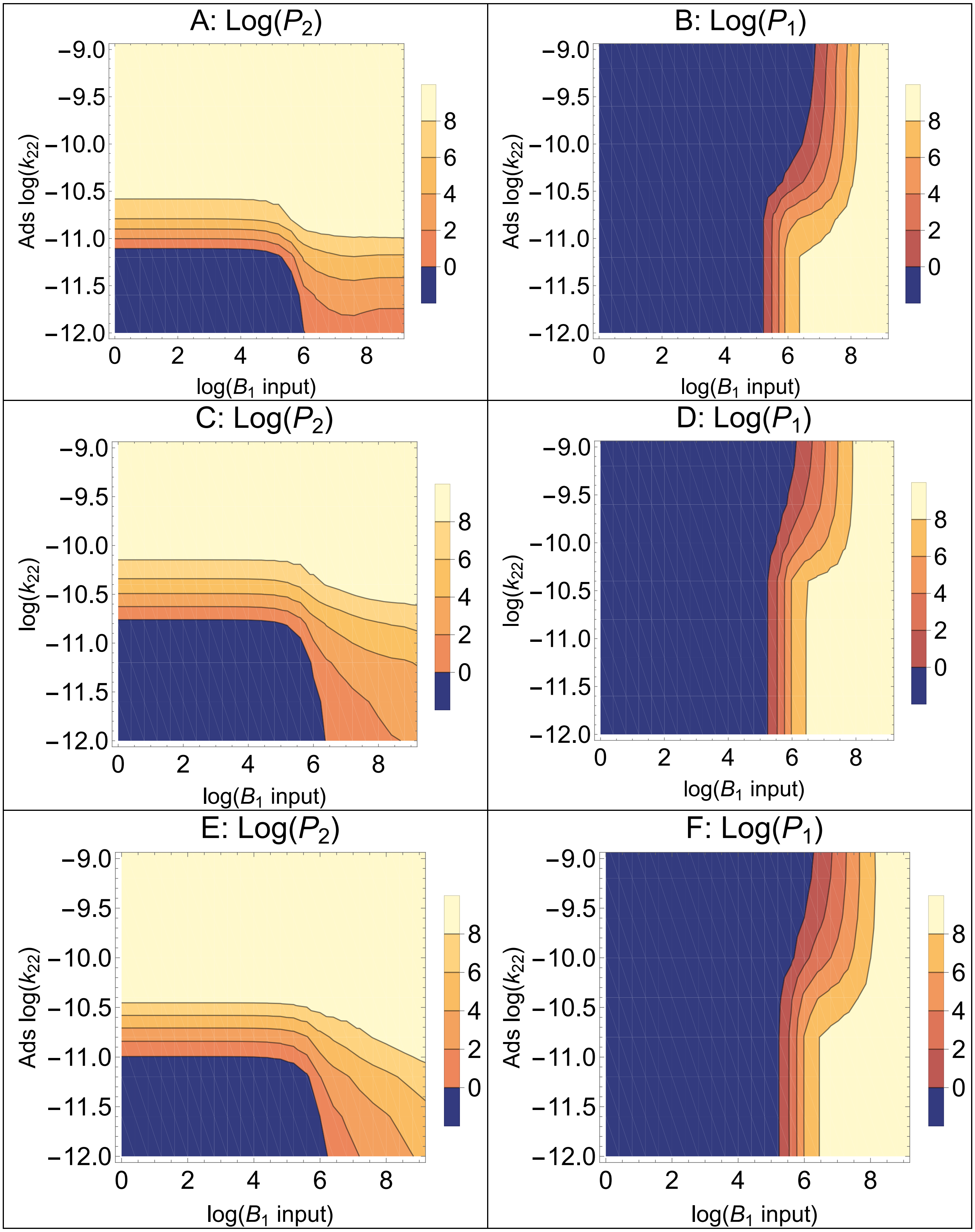

Fig. S1. (A) P_2 evolution with exponential distribution of lysis times (20 minute average), from text Fig. 1A. (B) P_1 evolution with exponential distribution of lysis times, from text Fig. 1B. (C) P_2 evolution with a gamma distribution of lysis times (20 minute average), using the same parameter values and initial

conditions as in (A). (D) P_1 evolution with a gamma distribution of lysis times. (E) P_2 evolution with a fixed lysis time of 20 minutes, using the same parameter values and initial conditions as in (A). (F) P_1 evolution with a fixed lysis time of 20 minutes. The main quantitative differences between the three methods lie in the position of the boundary between P_1 loss and P_1 ascent. The benefit of high B_1 input remains for all three methods.

Fig. S1 assumed transfer of free phage, infected cells, and uninfected cells. Such a protocol enables the eventual, approximate attainment of a stable age-of-infection distribution despite a fixed lysis time (Bull, Heineman and Wilke 2011). However, with cell densities declining in each culture due to phage killing (and especially differential killing of B_1 versus B_2), a stable distribution is not strictly attainable. Fig. S2 makes the same comparison but assumes transfer of just free phage. By transferring only free phage, each culture starts with high levels of synchrony, and with 50-minute cycles and the gamma distribution of lysis times or a fixed lysis time, even an approximate stable distribution is not expected. Not surprisingly, the discrepancies between the exponential and gamma are greater when transferring only free phage, but even in this case, the same qualitative conclusions apply as before. In contrast to Fig S1, the similarities in Fig S2 are greatest between the gamma and fixed lysis trials, which we interpret to result from the effect of transferring only free phage on disruption of a stable age-of-infection distribution. (The exponential distribution of lysis times should quickly decay to a stable age distribution.)

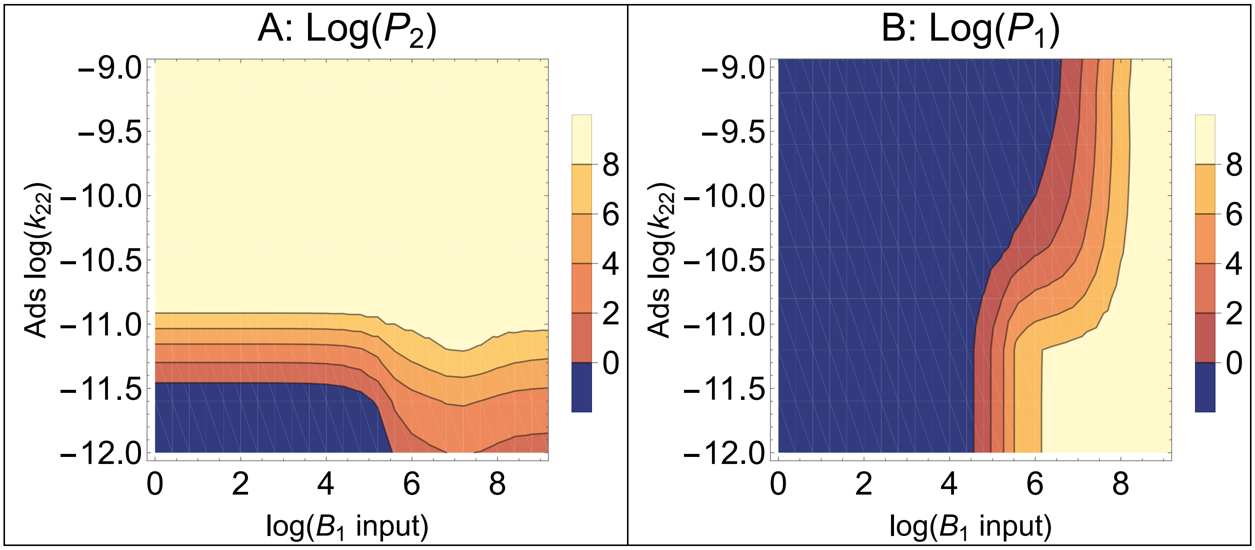

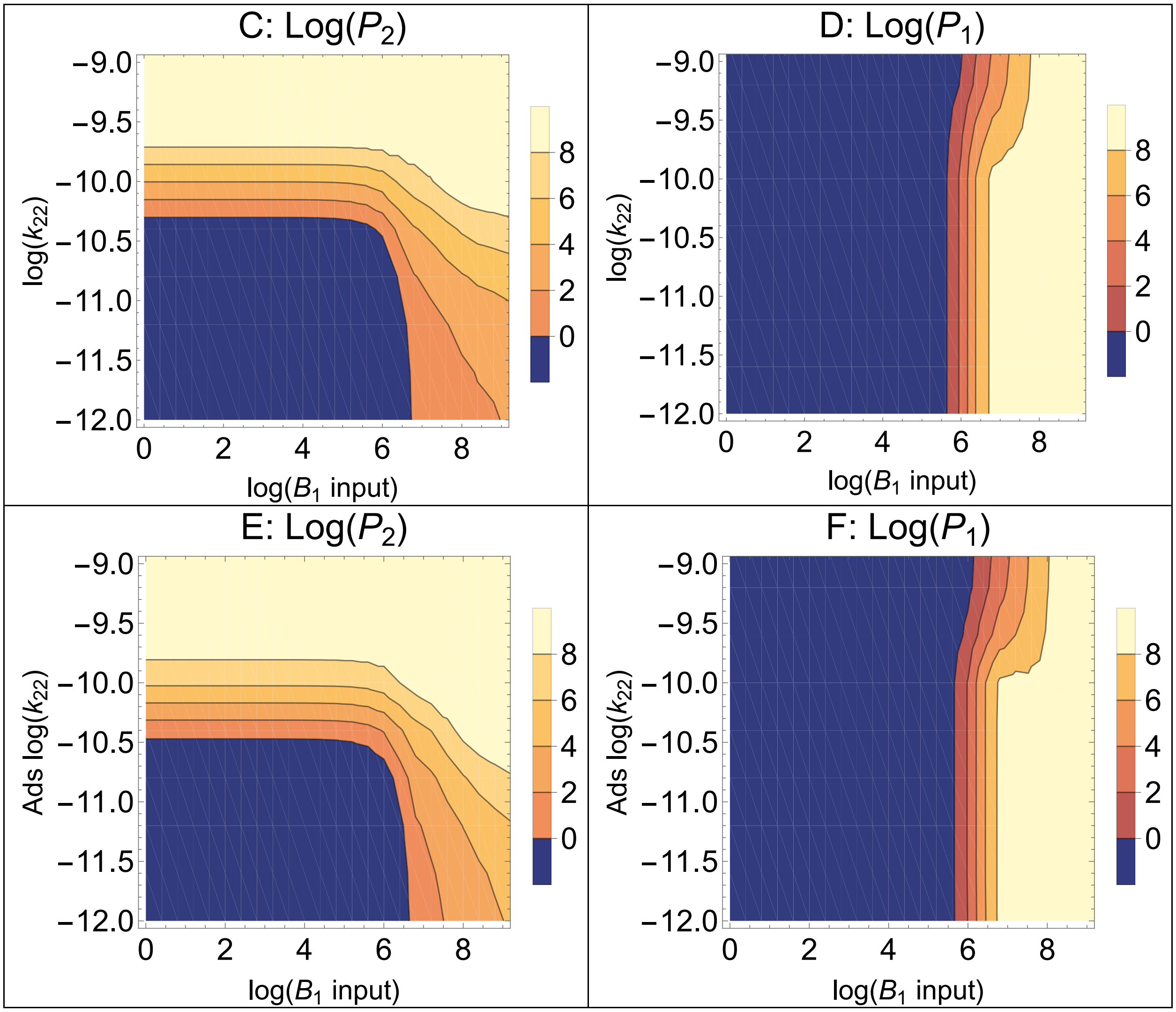

Fig. S2. The same trials as in Fig. S1, but assuming that only free phage are transferred between cultures; this change in protocol will introduce major deviations from a stable age distribution at the start of each cycle. (A) P_2 evolution with exponential distribution of lysis times (20 minute average). (B) P_1 evolution with exponential distribution of lysis times. (C) P_2 evolution with a gamma distribution of lysis times (20 minute average), using the same parameter values and initial conditions as in (A). (D) P_1 evolution with a gamma distribution of lysis times. (E) P_2 evolution with a fixed lysis time of 20 minutes, using the same parameter values and initial conditions as in (A). (F) P_1 evolution with a fixed lysis time of 20 minutes. The differences between the exponential and other two are greater than in Fig. S1, but a benefit of high B_1 input remains.

Again, at the level used in the present study, the protocols appear to be robust not only to the different possible models of lysis time but also to variations in how the transfers are made.

II. Additional protocols for Model 1 (adsorption block)

Serial transfer using Mixed or Sequential host presentation are not the only possible ways of adapting phages to a new host. One alternative is use of a continuous flow system, or chemostat (Borin *et al.* 2021). Another is a protocol known as Appelmans, which involves growth of phages on separate hosts followed by pooling the phages then again distributing aliquots of the phage pool to the separate hosts (Burrowes, Molineux and Fralick 2019; Bull, Wichman and Krone 2022). In Fig. S3, we briefly evaluate each of these for the case corresponding to Fig. 1A. The same patterns emerge as before. (A) and (B) assume continuous flow but vary in the rate of exchange (washout). The lower washout rate corresponds to a lower dilution rate under serial transfer. Although (B) shows more favorable mutant evolution than in Fig. 1A, the same pattern results from serial transfer (Mixed presentation) with a dilution of 0.25.

Fig. S3C shows mutant evolution for a variant of the Appelmans protocol (single cultures each using B_1 and B_2 as hosts, and adding undiluted portions of the phage pool to each at 0.1 dilution). Again, the pattern is broadly the same as with the other protocols.

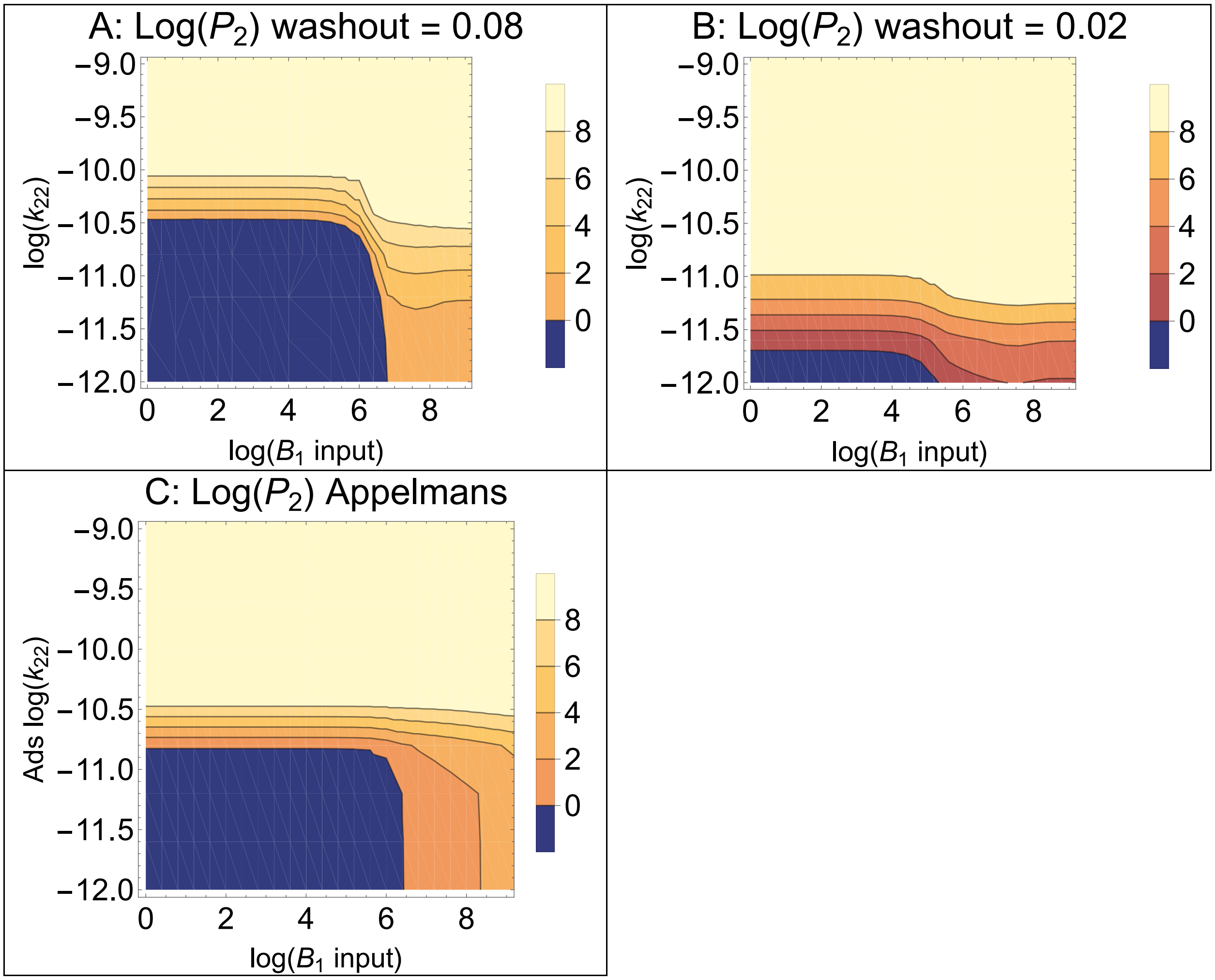

Fig. S3. (A) P_2 evolution with protocols other than serial transfer. (A) and (B) both apply continuous flow (chemostat), with different flow-through rates (washout rates, /min). The slower flow through enhances

mutant evolution just as does lower dilution with serial transfer. (C) is a form of the Appelmans protocol in which a phage pool is distributed to cultures of each pure host, phage are grown, pooled, and the process is repeated. The benefit of high B_1 input remains for all three methods.

References are provided in the main paper.
